# Local Equilibrium Approximation in Non-Equilibrium Thermodynamics of Diffusion

**DOI:** 10.3390/e27040400

**Published:** 2025-04-08

**Authors:** Kim R. Kristiansen, Bjørn Hafskjold

**Affiliations:** PoreLab, Department of Chemistry, Norwegian University of Science and Technology (NTNU), N-7491 Trondheim, Norway; kim.kristiansen@ntnu.no

**Keywords:** local equilibrium approximation, non-equilibrium entropy, diffusion, kinetic theory, molecular dynamics, telegrapher equation

## Abstract

Local equilibrium approximation (LEA) is a central assumption in many applications of non-equilibrium thermodynamics involving the transport of energy, mass, and momentum. However, assessing the validity of the LEA remains challenging due to the limited development of tools for characterizing non-equilibrium states compared to equilibrium states. To address this, we have developed a theory based on kinetic theory, which provides a nonlinear extension of the telegrapher’s equation commonly discussed in non-equilibrium frameworks that extend beyond LEA. A key result of this theory is a steady-state diffusion equation that accounts for the constraint imposed by available thermal energy on the diffusion flux. The theory is suitable for analysis of steady-state composition profiles and can be used to quantify the deviation from the local equilibrium. To validate the theory and test LEA, we performed molecular dynamics simulations on a two-component system where the two components had identical physical properties. The results show that deviation from the local equilibrium can be systematically quantified, and for the diffusion process we have studied here, we have confirmed that LEA remains accurate even under extreme concentration gradients in gas mixtures.

## 1. Introduction

The concept of a local equilibrium in non-equilibrium thermodynamics comes up in analyses of transport processes such as heat and mass transport. The term “local equilibrium” is not well defined [1]. Moreover, depending on whether a perturbation of an equilibrium state is temporal or spacial, the concept of local equilibrium in the perturbed state may have different meanings. Our use of the term is related to so-called linear non-equilibrium thermodynamics, where one assumes a local equilibrium, meaning that the Gibbs–Duhem equation is assumed valid with local values of the variables. The assumption is closely related to assuming that the local non-equilibrium entropy can be approximated by its equilibrium value in the entropy balance, which is the basis for finding the entropy production and coupled flux–force relations [2]. Whereas entropy is well understood and modeled for thermodynamic systems at equilibrium, the situation for systems out of equilibrium is less clear [3]. Usually, the assumption is introduced as an approximation and justified with successful consistency tests as “circumstantial evidence”. This is not satisfactory, and the extremely large forces and fluxes used in computer simulations that are necessary for acceptable signal-to-noise ratios in the results raise a need for a quantitative examination of local equilibrium approximation (LEA).

An alternative approach is to *assume the opposite, viz. a local non-equilibrium*, derive the corresponding theory whenever possible, and determine the deviation from the local equilibrium. A powerful criterion for this deviation is the difference in equilibrium and non-equilibrium entropy. Several theories for non-equilibrium entropy have been proposed for heat transport and diffusion, see, e.g., [4,5], and references therein. Cimmelli et al. [4] have given a very thorough discussion of four different theories and compared their pros and cons. They discuss the theories’ relation to kinetic theory, valid for low-density gases and conclude that in general, continuum theories that are compatible with kinetic theory are less universal, but more predictable than other theories.

In the present work, we pursue developing a theory for transport processes that do not depend on LEA. The theory is based on the Boltzmann equation and developed for diffusion in a binary gas mixture. It is then used to analyze results from molecular dynamics (MD) simulations of binary diffusion in a Lennard–Jones/spline (LJs) system [6]. We use an MD method introduced by Holian for diffusion simulations, which will be described in detail in Section 3. (Information about this method was communicated by B. L. Holian to W. G. Hoover in 1973, see Hoover and Ashurst [7] and Allen and Tildesley, p. 370 [8]. A very similar method has been used by Dong and Cao to compute self-diffusion coefficients [9]).

The two components are physically identical but they are identified as two types, “1” and “2” or “red” and “blue”. Therefore, the system’s properties are those of a one-component system, which simplifies the analysis and enables comparison with self-diffusion data computed with other methods. This is one of the simplest cases one can imagine for a study of a transport process. Our intention is to force the diffusion process as much as the system allows in an attempt to provoke a deviation from the local equilibrium. The results are compared with what we obtain from Fick’s law and with data from equilibrium simulations (mean square displacement).

According to Darken’s second equation [10], the mutual diffusion coefficient *D* in a binary mixture relates to the self-diffusion coefficients $D_{1}$ and $D_{2}$, as

(1) $$D=(x_{1}D_{2}+x_{2}D_{1})\Gamma \;.$$

where $x_{1}$ is the mole fraction of component 1 ($x_{2}=1-x_{1}$) and $\Gamma =\frac{1}{k_{B}T}{\left(\frac{\partial \mu_{1}}{\partial \ln x_{1}}\right)}_{P,T}$ is the thermodynamic factor. In our ideal case, Γ=1 and $D_{1}=D_{2}$, so that the diffusion coefficient we compute by generating a diffusive flux in the two-component system is simply the self-diffusion coefficient. This enables a comparison between the binary diffusion coefficient, generated with extreme fluxes and forces, and self-diffusion coefficients generated at global equilibrium.

## 2. Theory

### 2.1. Fick’s Law

In most common applications, diffusion is usually expressed in the form of Fick’s law, which states that the diffusion flux $J_{i}$ of component *i* is proportional to the gradient in the concentration $n_{i}$ of the corresponding component in the mixture

(2) $$J_{i}=-D\nabla n_{i}$$

where *D* is the diffusion coefficient (here, we use the flux of particles and the number density $n_{i}$ instead of the usual molar flux and molar concentration). Fick’s law for gas mixtures can be obtained ab initio from kinetic theory in the short mean free path limit, meaning that it holds for descriptions on length scales much longer than the mean free path. The divergence of Equation (2) at constant temperature and mass density, combined with the mass conservation law in a fluid at rest,

(3) $$\partial_{t}n_{i}+\nabla \cdot J_{i}=0\;,$$

yields the well-known diffusion equation, under the additional approximation that *D* is independent of $n_{i}$,

(4) $$\partial_{t}n_{i}=D\nabla^{2}n_{i}\;.$$

Fick’s law can also be obtained using the non-equilibrium thermodynamics methodology. Considering a binary mixture at isothermal conditions, the entropy differential is

(5) $$ds=-\frac{\mu_{1}}{T}dx_{1}-\frac{\mu_{2}}{T}dx_{2}=-\frac{\mu }{T}dx_{1}$$

where *s* is the entropy per particle, *T* the temperature, and $\mu_{i}$ and $x_{i}$ are the chemical potential and mole fraction of component *i*, respectively. We used $dx_{1}=-dx_{2}$ and introduced $\mu =\mu_{1}-\mu_{2}$. In order to keep the analysis relatively simple, we will in the following neglect the Dufour effect in the special case we consider here; in other words, we will neglect the heat flux accompanying diffusion. Using the mass conservation law, we obtain the entropy balance

(6) $$n\partial_{t}s=\frac{\mu }{T}\nabla \cdot J_{1}=\nabla \cdot \frac{\mu J_{1}}{T}-J_{1}\cdot \nabla \frac{\mu }{T}=-\nabla \cdot J_{s}+\sigma \;,$$

where *n* is the total number density. We have here identified the entropy flux $J_{s}=-\mu J_{1}/T$ and the entropy production rate $\sigma =J_{1}\cdot \left(-\nabla \mu /T\right)$. The latter is expressed on the standard form as a product of the flux $J_{1}$ and its conjugate driving force −∇μ/T. Assuming that this driving force is proportional to $J_{1}$ by some proportionality factor r>0 guarantees the positivity of the entropy-production rate, and gives the following relation at isothermal conditions:

(7) $$rJ_{1}=-\nabla \frac{\mu }{T}\Rightarrow J_{1}=-\frac{1}{rT}\frac{\partial \mu }{\partial n_{1}}\nabla n_{1}=-D\nabla n_{1}\Rightarrow r=\frac{\partial \mu /\partial n_{1}}{TD}\;.$$

The physical meaning of *r* is a resistivity relating the force (∇(μ/T)) to the flux (J). We arrive at Fick’s law by the general requirement that the flux is a linear combination of the thermodynamic driving forces in the system, where in this case −∇μ/T is the sole driving force. The identification of *D* from *r* clarifies the connection.

### 2.2. Generalized Diffusion Equation

While Fick’s law is adequate for describing diffusion processes when the system is everywhere in the local equilibrium, significant departures from the local equilibrium expose its unphysical features. For example, according to Fick’s law, a local change in the composition or flux anywhere in the system will *immediately* affect the evolution of the physical state everywhere else in the system. That is, Fick’s law propagates information across the spatial domain at an unphysical infinite speed, due to a chain of instantaneous molecular collisions arising as an artifact of neglecting the finite length of flight paths between collisions [11]. We seek a more general evolution equation of diffusion from the Boltzmann equation, which is not constrained by assumptions of a local equilibrium, and accounts fully for the discrete nature of molecular collisions, propagating information at speeds consistent with molecular velocities and collision dynamics. (Strictly speaking, this rectifies only one out of two mechanisms that violate causality. The resulting classical distribution of molecular velocities remains unbounded in momentum space, and so arbitrarily large velocities still have finite though vanishingly small probability amplitudes. A relativistic theory would be required to fully resolve the causality problem, but we stick to the classical formulation here.)

The momentum balance for the general case (several components with different properties, flowing system) was given by Snell et al. [12]. According to their work, the general equation of motion for component *i* in an isothermal multicomponent mixture, as obtained from the Boltzmann equation, is

(8) $$\partial_{t}\left(\rho_{i}u_{i}\right)=\nabla \cdot \left[A_{i}-B_{i}-\rho_{i}\left(u_{i}⊗u+u⊗u_{i}-u⊗u\right)\right]+x_{i}\sum_{k}\xi_{ik}x_{k}\left(u_{k}-u_{i}\right)-n_{i}\nabla \mu_{i}$$

where $\rho_{i}$ and $u_{i}$ are the mass density and the mean velocity of component *i*, respectively, u is the local barycentric velocity of the mixture, and $\xi_{ik}$ is a friction coefficient describing the exchange of momentum between components *i* and *k*. (The wotk by Snell et al. is based on the methodology used in the earlier works of Bearman and Kirkwood, where effective forces are introduced to create a pseudo-equilibrium state that emulates a true non-equilibrium state. Since the local state variables are, by construction, independent of the imposed forces, those state variables are assumed equal to the state variables in the local equilibrium)

The tensor $A_{i}$ denotes viscous contributions,

(9) $$A_{i}=x_{i}\sum_{k}\left[\left(\zeta_{ik}-\frac{2}{3}\eta_{ik}\right)I\nabla \cdot \left(x_{k}u_{k}\right)+\eta_{ik}\left(\nabla \left(x_{k}u_{k}\right)+{\left(\nabla (x_{k}u_{k})\right)}^{T}\right)\right]\;,$$

where $\zeta_{ik}$ and $\eta_{ik}$ are coefficients related to the bulk and shear viscosity, respectively. We refer the interested reader to [12] for their definitions. The tensor $B_{i}$ represents the traceless part of the diffusion momentum outer product (indicated by the symbol $\bar{⊗}$):

(10) $$B_{i}=\rho_{i}\bar{\left(u_{i}-u\right)⊗\left(u_{i}-u\right)}=\rho_{i}\left[\left(u_{i}-u\right)⊗\left(u_{i}-u\right)-\frac{1}{3}I\mathrm{Tr}\left[\left(u_{i}-u\right)⊗\left(u_{i}-u\right)\right]\right].$$

#### 2.2.1. Application to the Present System

When the two components are physically identical (which will be considered from here on), the viscosity coefficients $\zeta_{ik}$ and $\eta_{ik}$ in Equation (9) are independent of the pair ik. Moreover, when the fluid is stagnant, all contributions to $A_{i}$ that are proportional to ∇u vanish. Equation (8) can then be reduced to

(11) $$\partial_{t}\left(\rho_{i}u_{i}\right)+\nabla \cdot \left(\rho_{i}\bar{u_{i}⊗u_{i}}\right)=x_{i}\sum_{k}\xi_{ik}x_{k}\left(u_{k}-u_{i}\right)-n_{i}\nabla \mu_{i}\;.$$

We identify the fluxes $J_{i}=n_{i}u_{i}$. The difference between the equations of motion for the two components is

(12) $$\begin{array}{cc} \; & \partial_{t}\left(m_{1}J_{1}-m_{2}J_{2}\right)+\nabla \cdot \left(\frac{m_{1}\bar{J_{1}⊗J_{1}}}{n_{1}}-\frac{m_{2}\bar{J_{2}⊗J_{2}}}{n_{2}}\right)\\ \; & \;=2x_{1}x_{2}\xi \left(\frac{J_{2}}{n_{2}}-\frac{J_{1}}{n_{1}}\right)-n_{1}\nabla \mu_{1}+n_{2}\nabla \mu_{2}\;. \end{array}$$

where $m_{i}$ is the molecular mass of component *i* and the friction coefficients are symmetric, $\xi_{ik}=\xi_{ki}=\xi$. We now use that $J_{2}=-J_{1}$ and $\nabla \mu_{1}=\Gamma \frac{k_{B}T}{x_{1}}\nabla x_{1}$. Equation (12) can then be reduced to

(13) $$\bar{m}\partial_{t}J_{1}+\frac{\bar{m}}{n}\nabla \cdot \left[\bar{J_{1}⊗J_{1}}\left(\frac{c_{1}}{x_{1}}-\frac{1-c_{1}}{1-x_{1}}\right)\right]=-\frac{\xi J_{1}}{n}-\Gamma nk_{B}T\nabla x_{1}$$

where $\bar{m}=\left(m_{1}+m_{2}\right)/2$ is the average molecular mass, and $c_{1}=m_{1}/\left(m_{1}+m_{2}\right)$. In the system we consider here, $m_{1}=m_{2}=m$, so that c=1/2, which is used in the following. We see that under conditions where the terms on the left-hand side of Equation (13) are small, that is when temporal and spatial variations in the flux are small, we reproduce Fick’s law. This allows us to identify the friction coefficient ξ in terms of the diffusion coefficient:

(14) $$J_{1}=-\frac{\Gamma n^{2}k_{B}T}{\xi }\nabla x_{1}=-nD\nabla x_{1}\Rightarrow \xi =\frac{\Gamma nk_{B}T}{D}\;.$$

Using the product rule, we can split the term involving the divergence of the traceless kinetic energy tensor:

(15) $$\begin{array}{cc} \; & \nabla \cdot \left[\bar{J_{1}⊗J_{1}}\left(\frac{1}{x_{1}}-\frac{1}{1-x_{1}}\right)\right]\\ \; & =\left(\frac{1}{x_{1}}-\frac{1}{1-x_{1}}\right)\nabla \cdot \bar{J_{1}⊗J_{1}}-\left(\frac{1}{x_{1}^{2}}+\frac{1}{{\left(1-x_{1}\right)}^{2}}\right)\bar{J_{1}⊗J_{1}}\cdot \nabla x_{1}\;. \end{array}$$

Inserting Equation (15) into Equation (13), we find

(16) $$\begin{array}{cc} \; & \tau \left[\partial_{t}J_{1}+\frac{1}{2n}\left(\frac{1}{x_{1}}-\frac{1}{1-x_{1}}\right)\nabla \cdot \bar{J_{1}⊗J_{1}}\right]+J_{1}\\ \; & \;=-D\left[1-\frac{m}{2\Gamma n^{2}k_{B}T}\left(\frac{1}{x_{1}^{2}}+\frac{1}{{\left(1-x_{1}\right)}^{2}}\right)\bar{J_{1}⊗J_{1}}\cdot \right]\nabla n_{1} \end{array}$$

where we have identified the characteristic time $\tau =mn/\xi =mD/(\Gamma k_{B}T)$. We note that when the terms involving the divergence of the traceless kinetic energy tensor can be neglected, this reduces to

(17) $$\tau \partial_{t}J_{1}+J_{1}=-D\nabla n_{1}\;,$$

the divergence of which leads to the well-known telegrapher’s equation for $n_{1}$ when combined with Equation (3) for a stagnant fluid and gradients in *D* and τ are neglected. Equation (17) is a damped wave equation describing waves propagating with a characteristic speed $\sqrt{D/\tau }$ and attenuated over a characteristic time τ. Equation (2) corresponds to Equation (17) in the limit τ→0, which corresponds to a diverging wave speed that leads to the unphysically instantaneous propagation of information across the domain. The telegrapher’s equation takes into account the inertia of molecular motion that resists rapid changes to the diffusion flux, and its connection to non-equilibrium thermodynamics is thoroughly discussed in the work by Jou, Casas-Vázquez and Lebon [13]. There, the authors arrive at equations of the telegrapher type by means of the thirteen moment approximation, which is on the same order as Equation (16) with respect to the order of moments retained in the approximation to the full Boltzmann equation. Their work leads us to expect that the relationship between τ and *D* at this level of approximation should be corrected for higher order correlations in order to match real data, justifying an a priori unknown correction factor α such that the true relaxation time is ατ. The examples explicitly calculated by Jou et al. by continued fraction expansions yield constant α-values [13].

For the sake of simplicity, we will in the following restrict our attention to self-diffusion along a single dimension in three-dimensional space. Introducing also the correction factor α, Equation (16) is replaced by the one-dimensional equation for either component

(18) $$\alpha \tau \left[\partial_{t}J_{i}+\frac{1}{3n}\left(\frac{1}{x_{i}}-\frac{1}{1-x_{i}}\right)\nabla J_{i}^{2}\right]+J_{i}=-D\left[1-\frac{\alpha mJ_{i}^{2}}{3\Gamma n^{2}k_{B}T}\left(\frac{1}{x_{i}^{2}}+\frac{1}{{\left(1-x_{i}\right)}^{2}}\right)\right]\nabla n_{i}\;.$$

Rearranging Equation (18), we find that it can be written in the form

(19) $$\begin{array}{cc} r_{i}J_{i}= & -\left[1-\frac{\alpha mJ_{i}^{2}}{3\Gamma n^{2}k_{B}T}\left(\frac{1}{x_{i}^{2}}+\frac{1}{{\left(1-x_{i}\right)}^{2}}\right)\right]\frac{\nabla \mu_{i,\mathrm{eq}}}{T}-\frac{\alpha m}{nx_{i}T}\left[\partial_{t}J_{i}+\frac{1}{3n}\left(\frac{1}{x_{i}}-\frac{1}{1-x_{i}}\right)\nabla J_{i}^{2}\right]\;, \end{array}$$

with the identified resistivity $r_{i}=\left(\partial \mu_{i,\mathrm{eq}}/\partial n_{i}\right)/\left(TD\right)$, cf. Equation (7). The substitution of $\nabla n_{i}$ in Equation (18) with $\nabla \mu_{i}$ in Equation (19) was made by using $rD\nabla n_{i}=\frac{1}{TD}\frac{\partial \mu_{i,\mathrm{eq}}}{\partial n_{i}}D\nabla n_{i}=\frac{1}{T}\nabla \mu_{i}$. In Equation (19), we specified that the chemical potential $\mu_{i,\mathrm{eq}}$ is the equilibrium value, i.e., that obtained from the equation of state to distinguish it from the non-equilibrium value to be introduced below.

We can now identify the right-hand side of Equation (19) as a generalized driving force proportional to the flux, $X_{i}=r_{i}J_{i}$. In order to express the entropy production in terms of the flux–force pairs, we must identify a non-equilibrium entropy differential ds that reproduces the derived equation of motion. This can be achieved by considering the diffusion fluxes as independent non-equilibrium state variables in accordance with the extended irreversible thermodynamics framework [13].

(20) $$ds=-\frac{\mu_{1}}{T}dx_{1}-\frac{\mu_{2}}{T}dx_{2}+B_{1}dJ_{1}+B_{2}dJ_{2}$$

where $\mu_{i}$ is now a generalized non-equilibrium chemical potential of component *i*, and the $B_{i}$ are *a priori* unknown coefficients. In order to have correspondence with the equilibrium entropy, we must have that $\lim_{J_{i}\to 0}\mu_{i}=\mu_{i,\mathrm{eq}}$. Noting that the corresponding entropy balance equation takes the form

(21) $$\partial_{t}s+\nabla J_{s}=\sigma \;,$$

the connection with the equation of motion is made by assuming that the entropy production rate is a bilinear sum $\sigma =J_{1}X_{1}+J_{2}X_{2}$ with fluxes $J_{i}$ and forces $X_{i}$. That is, we have

(22) $$\begin{array}{cc} \sigma =-\sum_{i=1}^{2}\{J_{i} & \left[1-\frac{\alpha mJ_{i}^{2}}{3\Gamma n^{2}k_{B}T}\left(\frac{1}{x_{i}^{2}}+\frac{1}{{\left(1-x_{i}\right)}^{2}}\right)\right]\nabla \frac{\mu_{i,\mathrm{eq}}}{T}\\ + & J_{i}\frac{\alpha m}{nx_{i}T}\left[\partial_{t}J_{i}+\frac{1}{3n}\left(\frac{1}{x_{i}}-\frac{1}{1-x_{i}}\right)\nabla J_{i}^{2}\right]\}\;. \end{array}$$

We can now determine the coefficients $B_{i}$ by comparing this form of the entropy production rate to that which can be derived from the proposed entropy differential. We note that the contribution from the time derivative of $J_{i}$ to σ is

(23) $$\sigma \propto -\frac{\alpha mJ_{i}}{nx_{i}T}\partial_{t}J_{i}\;,$$

which allows us to deduce that

(24) $$n\partial_{t}s\propto -\frac{\alpha mJ_{i}}{nx_{i}T}\partial_{t}J_{i}\Rightarrow ds\propto -\frac{\alpha mJ_{i}}{n^{2}x_{i}T}dJ_{i}\Rightarrow B_{i}=-\frac{\alpha mJ_{i}}{n^{2}x_{i}T}\;.$$

Now, we use that $x_{1}+x_{2}=1$ to simplify Equation (20) to

(25) $$ds=-\frac{\mu }{T}dx_{1}+B_{1}dJ_{1}+B_{2}dJ_{2}$$

where $\mu =\mu_{1}-\mu_{2}$, equivalent to the definition in Equation (5). We can then obtain the $J_{i}$-dependence of μ through symmetry of mixed derivatives

(26) $$-\frac{\partial \mu /T}{\partial J_{i}}=\frac{\partial^{2}s}{\partial J_{i}\partial x_{1}}=\frac{\partial^{2}s}{\partial x_{1}\partial J_{i}}=\frac{\partial B_{i}}{\partial x_{1}}=-\frac{\alpha }{n^{2}T}\frac{\partial }{\partial x_{1}}\frac{mJ_{i}}{x_{i}}=\left\{\begin{array}{cc} \frac{\alpha mJ_{1}}{n^{2}x_{1}^{2}T} & i=1\\ -\frac{\alpha mJ_{2}}{n^{2}x_{2}^{2}T} & i=2 \end{array}\right.$$

Combined with the correspondence requirement to $s_{\mathrm{eq}}$ and that $J_{1}+J_{2}=0$, we find

(27) $$-\frac{\mu }{T}=-\frac{\mu_{\mathrm{eq}}}{T}+\int_{0}^{J_{1}}\;\;dJ^{\prime }\frac{\alpha mJ^{\prime }}{n^{2}T}\left(\frac{1}{x_{1}^{2}}+\frac{1}{{\left(1-x_{1}\right)}^{2}}\right)=-\frac{\mu_{\mathrm{eq}}}{T}+\frac{\alpha mJ_{1}^{2}}{2n^{2}T}\left(\frac{1}{x_{1}^{2}}+\frac{1}{{\left(1-x_{1}\right)}^{2}}\right)$$

We identify the second term on the right-hand side as the kinetic energy associated with species interdiffusion, multiplied by a factor α/T. This implies that in the non-equilibrium situation, the equilibrium chemical potential is replaced by a generalized chemical potential where a term proportional to the kinetic energy of diffusion is subtracted. This agrees with the assessment by de Groot and Mazur, who arrived at a similar generalized driving force by considering the necessity of subtracting the kinetic energy of diffusion from the typically defined internal energy to obtain the *truly* internal energy [14]. We may quantify the departure from the local equilibrium using the deviation of the local chemical potential from its equilibrium value:

(28) $$\mu_{\mathrm{eq}}-\mu =\frac{\alpha mJ_{1}^{2}}{2n^{2}}\left(\frac{1}{x_{1}^{2}}+\frac{1}{{\left(1-x_{1}\right)}^{2}}\right)=\alpha \left(\frac{mu_{1}^{2}}{2}+\frac{mu_{2}^{2}}{2}\right),$$

which reproduces the de Groot and Mazur result in the special case α=1. We restrict our attention to the steady-state equation of motion, where $\partial_{t}J_{1}=0$ and $\nabla J_{1}\propto \partial_{t}x_{1}=0$. This now reads

(29) $$J_{1}=-D\left[1-\frac{\mu_{\mathrm{eq}}-\mu }{\Gamma E_{k}}\right]\nabla n_{1}$$

where $E_{k}=3k_{B}T/2$ is the average molecular translational kinetic energy. Fick’s law with constant *D* predicts a constant composition gradient in steady state, according to Equation (4). According to Equation (29), we expect to see a curved composition profile when the local deviation in chemical potential from the local equilibrium value is significant relative to $\Gamma E_{k}$. The mechanism at play here is that of the diffusion process approaching its speed limit dictated by the local distribution of molecular velocities. Whereas a Fickian model would violate this speed limit by allowing the diffusion flux to increase beyond the rate of transport permissible by the ballistic motion of molecules at a given temperature, this generalized formulation properly enforces said speed limit in a continuous and physically consistent manner. Investigating the curvature of the steady-state composition profile resulting from a large imposed diffusion flux allows us to properly quantify the deviation from the local equilibrium.

#### 2.2.2. The Non-Equilibrium Entropy

We note that for fixed $x_{i}$, we can use the result for $B_{i}$, Equation (24), in Equation (20) and integrate to give

(30) $$s-s_{\mathrm{eq}}=-\sum_{i}\frac{\alpha \;mJ_{i}^{2}}{2n^{2}x_{i}T}=-\frac{\alpha mJ_{1}^{2}}{2n^{2}T}\left(\frac{1}{x_{1}}+\frac{1}{1-x_{1}}\right)=-\frac{\alpha mJ_{1}^{2}}{2n^{2}x_{1}\left(1-x_{1}\right)T}\;,$$

which shows that the non-equilibrium entropy *s* is always smaller than the equilibrium value, which is consistent with the second law. This form of the non-equilibrium entropy is also in agreement with that derived by Jou et al. [13], which means that the nonlinear contribution to the flux, not included in the theory of Jou et al., does not directly influence the value of the local entropy. The absolute deviation in the entropy from the local equilibrium value has a minimum at $x_{1}=0.5$.

## 3. MD Simulations

**Figure 1:**
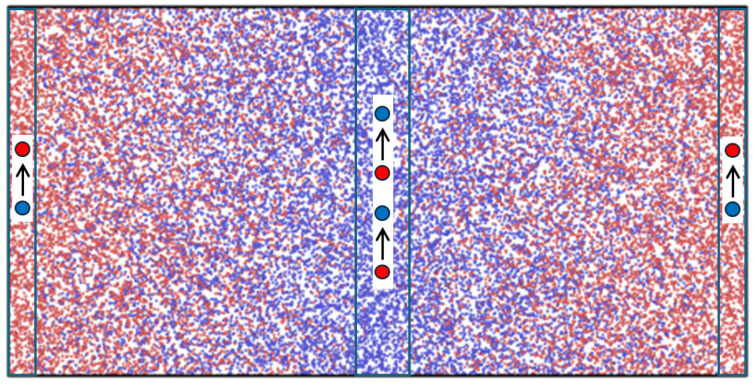
Layout of MD box used in the simulations. The particle types are swapped as indicated in the end and central regions of the MD box (the swapping layers).

Non-equilibrium molecular dynamics (NEMD) simulations were carried out to provide data for use with the theory. The layout of the MD system is shown in Figure 1.

The system consisted of N=32,768 Lennard–Jones/spline (LJs) particles [6] in a rectangular cuboid with aspect ratio $L_{x}:L_{y}:L_{z}=2:1:1$. The LJs potential is a smoothly truncated Lennard–Jones potential with cut-off distance $r_{c}\approx 1.7\sigma$. The MD box had periodic boundary conditions in all three directions. The box was divided into 64 layers of equal thickness in the *x*-direction for recording local data of temperature, density, composition, etc. The particles were of two types, 1 (“red”) and 2 (“blue”), with exactly the same mass and potential parameters [8].

Concentration gradients and diffusive mass fluxes were generated with an in-house NEMD code using the MEX algorithm [1]. The MEX algorithm works so that a randomly picked particle of type 2 is changed to type 1 in one of the control volumes at the box ends (see Figure 1) while a particle of type 1 is changed to type 2 in the central volume at the same time. The overall concentration was thus kept constant. The identity swapping was tried at regular intervals, which varied between every time step and every 100th time step. The swapping acts as source and sink terms at the boundaries (ends and center of the MD box). At steady state, the mass flux in the bulk fluid is related to the swapping frequency, ν=numberofswaps/time, $J_{i}=\nu_{i}/2A$, where *A* is the cross-sectional area of the box. By changing the swapping frequency, the diffusion flux was controlled from zero (equilibrium) to maximum (saturated control volumes, i.e., $x_{1}=1$ at the box ends and $x_{1}=0$ in the box center). If there were no candidate particles to swap in either control volume, the swapping was skipped and the actual swapping frequency was reduced accordingly. The reported mass fluxes in Table 1 were computed from $J_{1}=\langle n_{1}u_{1}\rangle$ for each layer in the fluid and then averaged over the layers in the bulk. The logged swapping frequency was used for consistency check. Typical swapping frequencies varied from 9 to 22 (in LJ units).The mass current is half the swapping frequency (due to the fact that particles move both left and right from the swapping layers), and the mass flux is the current divided by the cross-sectional area. For the results reported here, the time between each swap was on average between 0.02 and 0.06, which is three orders of magnitude shorter than the characteristic time $\tau =mD/(\Gamma k_{B}T)\approx 10$. We can therefore expect that the non-continuous particle swapping at the boundaries leads to a mass flux that is smooth in time (which was also observed).

The simulations started from an FCC lattice configuration with $N_{1}=N_{2}=N/2$. To prevent heating of the system due to the entropy production, the system temperature was controlled by thermostating each of the 64 layers to the same set temperature and constant density was assured by rapid equilibration of the pressure in the fluid. The overall density was controlled by the total number of particles in the fixed box volume. The thermostat was a simple velocity scaling and shifting subject to set the local momentum to zero [1]. The scaling factor was the same in *x*-, *y*-, and *z*-directions, but the shift was direction specific. The thermostat was activated every 20 time steps.

One may argue that enforcing a constant temperature through the system has an impact on properties derived from temperature gradients such as Soret and Dufour effects. This should be considered for systems where the components do not have identical properties, but for the present system, such coupled effects vanish.

Six cases were generated with the density and temperature as listed in Table 1. All values of physical properties reported here are in Lennard–Jones units. The mean free path, λ, was estimated from elementary kinetic theory as $\lambda =1/(\pi \sqrt{2}n)$ and compared with the half box length, $L_{x}/2$. Case 1 is a very dilute (ideal) gas which is not well suited for MD simulations due to the fact that the particles are mostly in free flight; the mean free path is of the same order as $L_{x}/2$ (the distance between the swapping layers) in this case. Nevertheless, this low-density case was included here because of the kinetic theory basis for the analysis. Case 6 is close to the LJs system’s dew point (at n=0.026 for T=0.7). When the mean free path is much larger than the range of the potential, particles may overlap at the end of a time step. To avoid strong accelerations if this happens, the particles were given a reflecting “shield” at $r_{ik}=0.8$.

A total of 4,000,000 time steps of length δt=0.002 in Lennard–Jones units were used to produce data for analysis. The final 2,000,000 time steps were used to represent steady state with J=0.

## 4. Results and Discussion

**Figure 2:**
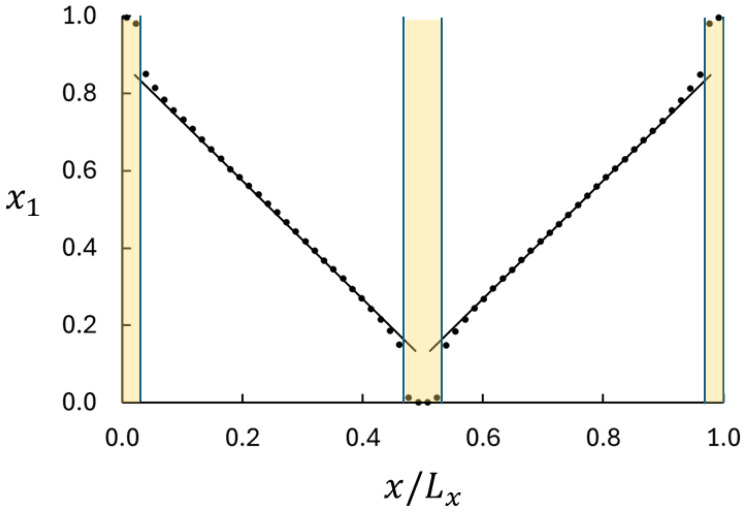
Simulation results for mole fraction profile for Case 2. The straight lines are fitted to the central part of the profiles ($0.15<x/L_{x}<0.35$ and $0.65<x/L_{x}<0.85$) and used to estimate the Fick’s law diffusion coefficient from Equation (2). The positions of the swapping layers are marked yellow. The jumps in $x_{1}$ near the ends and in the center of the MD box occur between the swapping layers and the bulk. Note the slight curvatures of the profile in the bulk near the boundaries. The curvature signifies deviation from the local equilibrium and is the basis for analysis with Equation (29). The profiles for the other cases are qualitatively the same as shown here.

We shall now analyze the binary diffusion data using the theory developed in Section 2.2. Central in this analysis is the curvature of the mole fraction profile and the deviation from the local equilibrium. A typical mole fraction profile is shown in Figure 2. The following are three important features: (i) the profile appears to be linear in the central part of the bulk regions, (ii) the profile is slightly curved near the ends of the bulk regions, and (iii) there are significant jumps in $x_{1}$ between the swapping volumes and the bulk. We shall focus on the profile in the bulk near the boundaries where the profile is slightly curved. Equation (29) shows that for $J_{1}$ and *D* to be constant in the bulk region, the fraction $\left(\mu_{\mathrm{eq}}-\mu \right)/\Gamma E_{k}$ must follow from

(31) $$\frac{\mu_{\mathrm{eq}}-\mu }{\Gamma E_{k}}=1+\frac{constant}{\nabla x_{1}}$$

where $constant=J_{1}/\left(nD\right)>0$ and $\nabla x_{1}<0$. In other words, there is a direct relation between the nonlinearity in the mole fraction profile and $\mu_{\mathrm{eq}}-\mu$. Hence, we use the curvature in the mole fraction profile to analyze the deviation from the local equilibrium.

The jumps in $x_{1}$ are consequences of the large imposed mass flux and the resolution of the data acquisition in the *x*-direction. Similar jumps are seen for temperature profiles in simulations of heat transport [15,16]. A weaker imposed mass flux would bring the system closer to equilibrium and show smaller jumps, but the objective here is to bring the system as far from equilibrium as possible. Clearly, the maximum difference in composition, and therefore the maximum mass flux, is obtained when the swapping layers are saturated with one of the components.

A central question in this work is to what extent LEA is valid. Equation (28) gives a direct measure of the deviation from the equilibrium chemical potential. We see that $\mu_{\mathrm{eq}}-\mu$ is positive. From Equation (29), we see that Fick’s law will always underestimate the value of *D*. The difference can be quantified with the second term in the square bracket in Equation (29). If the fraction $\left(\mu_{\mathrm{eq}}-\mu \right)/\Gamma E_{k}$ is small compared to 1, the local equilibrium is a good approximation, reducing Equation (29) to Fick’s law. Otherwise, the approximation is poor. Exactly what we mean by an acceptable approximation is a matter of choice, but the important point is that whatever choice is made, it can be quantified and communicated. Equation (28) shows that the deviation from equilibrium depends on $x_{1}$ with the minimum value at $x_{1}=0.5$. The minimum value of $\left(\mu_{\mathrm{eq}}-\mu \right)/\Gamma E_{k}$ is then $8\alpha mJ^{2}/\left(3n^{2}kT\right)$ with Γ=1.

**Figure 3:**
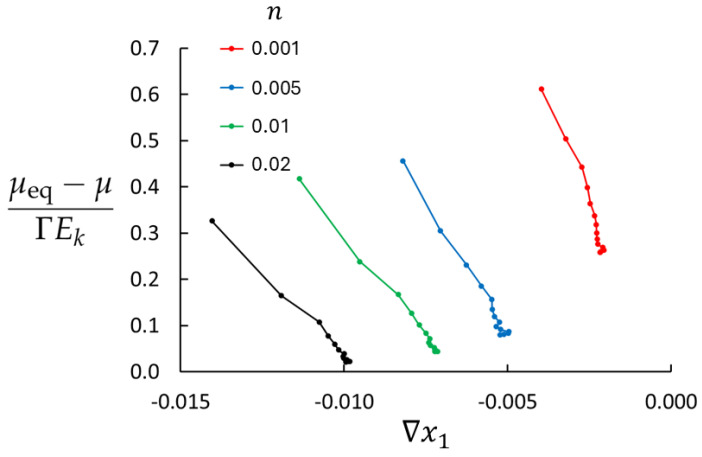
Simulation results for the deviation from the local equilibrium according to Equations (28) and (29) for four different densities (Case 1, 2, 3, and 6). All cases have T=0.7.

Lack of local equilibrium will depend on density, temperature, and the mass flux. Figure 3 shows the deviations from the local equilibrium for four densities used in this study at T=0.7 and maximum imposed mass flux (saturated control volumes). The right endpoint of each graph represents the minimum value of $|\nabla x_{1}|$, found in the central part of the bulk region where the system is closest to equilibrium. The maximum value of $|\nabla x_{1}|$ is the left endpoint (near the swapping layers) where the deviation from equilibrium is largest. LEA appears to be very poor for the lowest density, at best approximately 25% (in the central part of the bulk) and worse towards the swapping layers. In this region, the diffusive velocity of the rare component is large, $u_{i}=J_{i}/n_{i}$, and the right-hand side of Equation (28) becomes large. Furthermore, for n=0.001, molecular collisions are rare and mass transport is to a large extent ballistic. The situation is much better for higher densities. For n=0.02, the deviation from equilibrium is approximately 2 % in the central part of the bulk, rising to about 30 % near the swapping layers.

**Figure 4:**
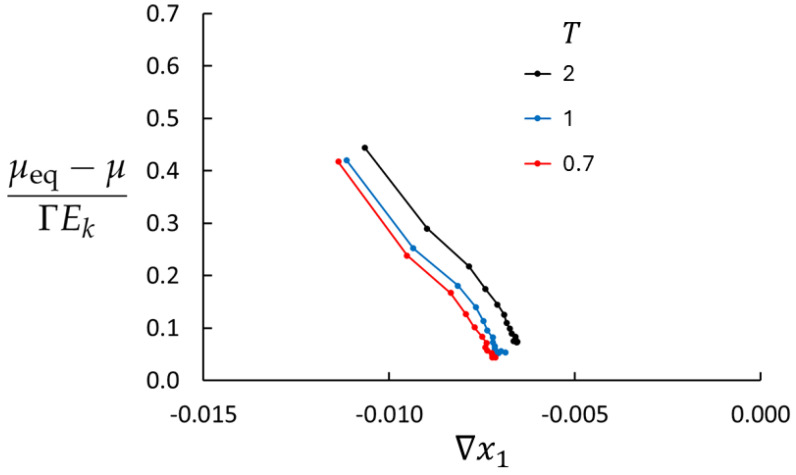
Simulation results for the deviation from the local equilibrium according to Equations (28) and (29) for three temperatures (Case 3, 4, and 5). All cases have n=0.01.

Figure 4 shows the deviations from the local equilibrium for three temperatures (T=0.7,1.0, and 2.0), n=0.01, and maximum mass flux. LEA is less than 4% off in the central part of the bulk for the highest temperature and better for the lower temperatures.

**Figure 5:**
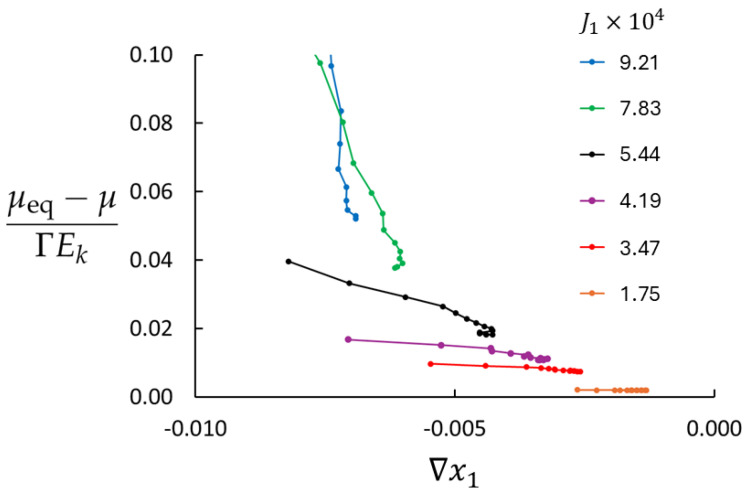
Deviation from the local equilibrium according to Equations (28) and (29) for six cases with different imposed mass fluxes. All results are for n=0.01 and T=0.7 (Case 4). Note that the graphs for the two highest mass fluxes are incomplete; top values are larger than the axis maximum value.

LEA will clearly be better for reduced mass flux. According to Equations (28) and (29), the $\left(\mu_{\mathrm{eq}}-\mu \right)/\Gamma E_{k}$ is proportional to $J_{1}^{2}$. We simulated Case 4 for six additional imposed fluxes. The results are shown in Figure 5. In addition to the reduced value of $\left(\mu_{\mathrm{eq}}-\mu \right)/\Gamma E_{k}$ with reduced flux, it is worth noting that the range spanned by $\nabla x_{1}$ is also reduced with reduced flux, meaning that the mole fraction profile is less curved.

**Figure 6:**
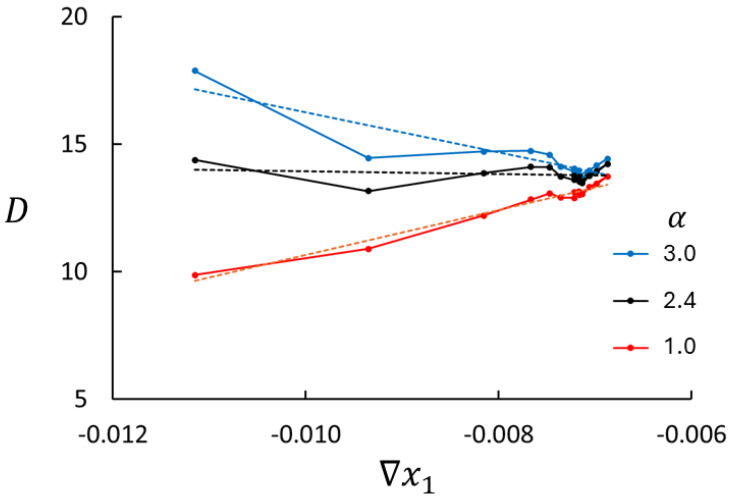
The diffusion coefficient as function of mole fraction for three different values of α, n=0.01 and T=0.7 (Case 4). The dashed lines are fitted linear functions to the MD data.

The parameter α in Equation (28), which is implicit in Equation (29), was determined as follows. In principle, *D* may be a function of $x_{1}$ and $\nabla x_{1}$. However, the system is an ideal mixture of particles with identical physical properties at uniform density and temperature. The diffusion coefficient in Equation (29) must therefore be independent of $x_{1}$ and $\nabla x_{1}$. The value of α was adjusted to satisfy this condition. An example of the relation between α and *D* is shown in Figure 6. A value of α that is too large (small) gives too much (little) emphasis on the curvature in $\nabla x_{1}$, especially near the ends of the mole fraction range (cf. Figure 2). By fitting a linear function to *D* vs. $\nabla x_{1}$, α was determined as the value giving zero slope (=2.4 for Case 4 shown in Figure 6). We note that the value of *D* for the largest values of $\nabla x_{1}$, where the $x_{1}$-profile is linear, is not very sensitive to the value of α. This procedure gave almost the same value for α for the six cases studied here (Table 2).

We note that the condition on *D* applies to the special case of identical components, in a more general case one must expect that *D* depends on the composition.

Numerical values for the diffusion coefficient obtained from Equation (29) for the six cases are shown in Table 2. The coefficient obtained from Fick’s law is consistently smaller than *D* and the agreement between $D_{\mathrm{Fick}}$ and *D* is better for higher densities with fixed temperature and for higher temperature with fixed density. Both trends can be understood in terms of the system’s ability to dissipate the effect of the perturbation.

As mentioned in the Introduction, the main purpose of this work is to develop a tool for analysis of LEA in non-equilibrium thermodynamics. We considered a very special case, mutual diffusion of an ideal system in gas phase, and used kinetic theory as the basis. As it stands, the method has limited value for higher densities, which requires an improved version of the kinetic theory. Another limitation is that we approximated Γ, $\mu_{i}$, and $s_{\mathrm{eq}}$ with ideal-gas values. These properties must be determined from a more realistic equation of state, which is available for the LJs model [17].

The theory was developed for binary diffusion at low density in general and simplified for a system with identical components. Application to binary systems with different components is therefore possible. For instance, analysis of mutual diffusion in a binary mixture with two different components will be possible with the same tool, although it will require more information to evaluate the terms $A_{i}$ and $B_{i}$ in Equations (9) and (10). The conclusions with respect to LEA may or may not be the same as we draw in this work, but the point is that the theory presented here can be used for the analysis. The results presented in Table 2 suggest that increased density will improve LEA due to more frequent molecular collisions.

Another interesting application is heat transport, where the same considerations of the combination of ballistic and diffusive transport mechanisms apply.

Equation (30) gives us a tool to compute the difference between the non-equilibrium and equilibrium entropy. We found for $\left(s-s_{\mathrm{eq}}\right)/s_{\mathrm{eq}}$:

(32) $$\frac{s-s_{\mathrm{eq}}}{s_{\mathrm{eq}}}=-\frac{\alpha m}{2\;s_{\mathrm{ST}}\;n^{2}x_{1}\left(1-x_{1}\right)T}J_{1}^{2}$$

where we used the Sackur–Tetrode ideal gas entropy, $s_{\mathrm{ST}}$, as representing $s_{\mathrm{eq}}$. Using Case 2 as an example and assuming that the LJs model represents argon with the potential parameters, $\epsilon /k_{B}=120$ K and σ=0.34 nm, we find that the molar equilibrium entropy is 125 J mol^−1^ K^−1^ and the difference between the molar non-equilibrium and equilibrium entropies is 0.5 J mol^−1^ K^−1^ for $x_{1}=0.5$. The relative deviation is $\left(s-s_{\mathrm{eq}}\right)/s_{\mathrm{eq}}=0.004$ for $x_{1}=0.5$ and 0.01 for $x_{1}=0.9$ (the extreme value in the bulk, *cf* Figure 2). These results are additional demonstrations of the excellence of LEA in this diffusion case.

The importance of the kinetic energy of diffusion [14] can be illustrated by the ratio between the kinetic energy of diffusion and the thermal energy,

(33) $$\mathrm{ratio}=\frac{m}{3k_{B}T}{\left(\frac{J_{1}}{n_{1}}\right)}^{2}\left(\frac{1}{x_{1}^{2}}+\frac{1}{{\left(1-x_{1}\right)}^{2}}\right)\;.$$

Again using Case 2 as an example, we found that the ratio is 0.04 and 0.46 for $x_{1}=0.5$ and $x_{1}=0.9$, respectively. This result shows that even if the kinetic energy of diffusion is large compared with the thermal energy, the deviation from the local equilibrium is small and LEA is good.

A more detailed analysis of the velocities should include the velocity distributions. A local Maxwellian distribution would indicate a local equilibrium. An analysis of the velocity part of the Boltzmann *H*-function can be used to quantify the deviation from equilibrium [1]. The velocity distributions can also be used for a discussion of the local temperature and the role of the kinetic energy of diffusion in computing the local temperature, which we have not considered here. Based on the thermodynamic results we have, which show that the system is close to equilibrium, we expect that the velocity distribution for each component will be Gaussian in the *y*- and *z*-directions, a shifted Gaussian in the *x*-direction, and Gaussians with zero mean for the combined distributions of the two components in all three directions. The variance of the distributions will give us the local kinetic temperature.

## 5. Conclusions

Using a theoretical framework based on the Boltzmann equation, we quantitatively assessed local equilibrium approximation (LEA) commonly used in non-equilibrium thermodynamics. Our analysis, which applies to both equilibrium and non-equilibrium conditions, was developed for an ideal diffusion process in a binary system composed of two physically identical components. This approach yields a nonlinear generalization of the telegrapher’s equation. In particular, the steady-state equation we derive, Equation (29), provides a novel method for determining a corrected relaxation time from steady-state measurements, which are generally easier to analyze than transient phenomena. We then employ the relaxation time correction factor, α, to quantify the deviation from equilibrium by comparing the equilibrium entropy with its non-equilibrium counterpart, Equation (30). This framework offers a quantitative measure of the deviation from the local equilibrium and enhances the assessment of LEA in non-equilibrium thermodynamics.

Our results indicate that LEA holds well for most conditions except at the lowest density examined (0.001 in Lennard–Jones units). At low density, a large mean free path and a high diffusion kinetic energy relative to the system’s thermal energy lead to significant deviations from the local equilibrium. In contrast, for higher gas-phase densities, LEA remains a robust approximation for the system considered. Notably, even under conditions of large fluxes and forces where, for argon, the mass fluxes and concentration gradients reach approximately 10^4^ mol m^−1^ s^−1^ and 10^10^ mol m^−4^, respectively (Case 2), the estimated diffusion coefficient for argon at about 1 bar and 85 K is on the order of 10^−6^ m^2^ s^−1^, which agrees well with experimental data [18].

## Figures and Tables

**Table 1:** Thermodynamic states and imposed mass flux used in this work. All values are in Lennard–Jones units. The values of $|\nabla x_{1}{|}_{\max}$ are the maximum difference in $x_{1}$, 1.0, divided by $L_{x}/2$. The actual values obtained in the simulations are given in Table 2.

Case	*n*	*T*	$L_{x}/2$	$|\nabla x_{1}{|}_{\max}\times 10^{3}$	λ	$J_{1}\times 10^{4}$
1	0.001	0.7	254	3.9	225	1.780±0.005
2	0.005	0.7	148	6.8	45.0	4.88±0.02
3	0.01	0.7	118	8.5	22.5	6.64±0.01
4	0.01	1.0	118	8.5	22.5	9.136±0.008
5	0.01	2.0	118	8.5	22.5	16.09±0.01
6	0.02	0.7	94	10.6	11	10.17±0.03

**Table 2:** Simulation results for the six cases. The diffusion coefficient $D_{\mathrm{Fick}}$ and *D* were determined from Equations (2) and (29), respectively. The values for $\nabla x_{1}$ represent the central part of the mole fraction profiles in Figure 2, used to determine $D_{\mathrm{Fick}}$. The values of $D_{\mathrm{MSD}}$ were determined in independent equilibrium simulations. For the α-values, please see the text.

Case	$\nabla x_{1}\times 10^{3}$	$D_{\mathrm{Fick}}$	*D*	$D_{\mathrm{MSD}}$	α
1	−2.19±0.03	81±1	114±2	109.7±0.5	2.1
2	−5.16±0.05	18.9±0.1	20.8±0.2	21.73±0.05	2.2
3	−7.28±0.03	9.1±0.1	9.63±0.05	10.73±0.03	2.6
4	−7.10±0.05	12.87±0.04	13.8±0.1	14.77±0.05	2.4
5	−6.66±0.04	24.20±0.02	26.4±0.1	27.5±0.1	2.1
6	−9.95±0.03	5.11±0.03	5.27±0.02	5.19±0.01	2.2

## Data Availability

Data used in this study are available on request to the corresponding author.
